# Ultrasonographic documentation of type-3 abomasal ulcer in a cow with left displacement of the abomasum

**DOI:** 10.1186/s13028-020-00527-1

**Published:** 2020-06-12

**Authors:** Christian Gerspach, Carina Oschlies, Jasmin Kuratli, Ueli Braun

**Affiliations:** 1 Department of Farm Animals, Vetsuisse Faculty, University of Zurich, Zurich, Switzerland; 2 Institute of Veterinary Pathology, Vetsuisse Faculty, University of Zurich, Zurich, Switzerland

**Keywords:** Abomasum, Cattle, Type-3 abomasal ulcer, Ultrasonography

## Abstract

**Background:**

Ultrasonographic documentation of perforated abomasal ulcer has not been published till now. This report describes the clinical, ultrasonographic and postmortem findings in a Jersey cow with type-3 abomasal ulcer and left displacement of the abomasum (LDA).

**Case report:**

The main clinical findings were abnormal demeanour, rumen atony and tympany, positive foreign body tests, positive auscultation and simultaneous ballottement on the left side, abdominal guarding and loss of negative pressure in the abdominal cavity. The tentative diagnosis was peritonitis and LDA. Abdominal ultrasonography produced images typical of LDA, and in one location between the abdominal wall and abomasum there was a layer of fibrin, a fibrin clot, a break in the abomasal contour, suggestive of a perforated ulcer, and partial obstruction of this gap with fibrin. The diagnosis of perforated abomasal ulcer with subsequent peritonitis was confirmed during postmortem examination.

**Conclusions:**

The examination of this case shows that under certain circumstances, ultrasonographic imaging of a perforated abomasal ulcer in a cow is possible. Antemortem diagnosis of type-3 abomasal ulcer is preferable to relying on exploratory laparotomy and/or post-mortem examination.

## Background

Abomasal ulcers in cattle are classified as types 1–5 (U1–U5) [1–5]. U1 is a non-perforated ulcer of the abomasal mucosa associated with minimal haemorrhage [1], whereas U2 involves the erosion of a large blood vessel and is therefore associated with massive intraluminal haemorrhage [2]. Type-3 is a perforated ulcer accompanied by localised peritonitis [3], U4 is a perforated ulcer characterised by generalised peritonitis [4] and U5 is an ulcer that has perforated into the omental bursa causing omental bursitis [5]. Ultrasonographic examination of the abomasum has been described in cows [6] as well as in calves [7]. The abomasum can be visualised about 10 cm caudal to the xyphoid in cows and it occupies more space on the right side of the median than the left. The abomasal wall is difficult to consistently visualize but when seen appears as a thin echogenic line. Abomasal contents appear heterogeneous with moderately echogenic material interspersed with echogenic stippling. Abomasal folds are occasionally seen as echogenic structures. With left and right displacement, the abomasum can be visualised between the left abdominal wall and rumen [8–10] and between the right abdominal wall and the liver [11], respectively. In a study of cows with right displacement of the abomasum and cows with abomasal volvulus it was not possible to differentiate the two conditions on the basis of ultrasonographic findings [11]. Likewise, ultrasonographic visualisation of abomasal ulcers has not been possible so far. It may be possible to visualise abomasal ulcers using high-resolution ultrasound probes, but the main reason for not being able to detect them via ultrasonography is the fact that ulcers are usually not located directly adjacent to the abdominal wall and the depth of typical abomasal ulcers preclude imaging with traditional ultrasonographic machines [12]. However, a tentative diagnosis can sometimes be made indirectly based on ultrasonographic findings; omental bursitis is strongly suggestive of type-5 abomasal ulcer [5] and generalised peritonitis is suggestive of type-4 ulcer [4]. Other abomasal disorders that have been diagnosed sonographically include malignant lymphoma [13] and a metal foreign body in the abomasum of a cow [14]. The present report is apparently the first to describe a cow with type-3 abomasal ulcer in which the perforated ulcer as well as the inflammatory lesions caused by the ulcer could be imaged ultrasonographically.

## Case presentation

A 2.3-year-old Jersey cow that had calved 4 weeks previously without complications developed acute mastitis 2 weeks after parturition followed by unilateral peritarsitis 2 weeks later. Treatment with oxytetracycline (30 mg, intravenously (IV)) led to transient improvement but the cow relapsed and stopped eating. Treatment with a rumen magnet, glucose infusion (500 mL of a solution containing 109 g glucose and 70 g sorbitol), brotizolam (1 mg, IV) and dexamethasone (20 mg, IV) was unsuccessful. The cow developed rumen tympany and spontaneous grunting and was therefore referred to our clinic.

The general health and appetite of the cow were poor, the skin surface temperature and muzzle were cold to the touch and the scleral vessels were congested. The heart rate was 84 beats/min (normal 60–80 beats/min, [15]), the respiratory rate was 40 breaths/min (normal 15–35 breaths/min, [15]) and the rectal temperature was 39.0 °C (normal 38.6–39.0 °C, [15]). There was rumen atony and tympany.

The cow showed signs consistent with abdominal pain (grunting in response to foreign body tests, which included the whithers pinch test, pain percussion of the reticular area and the pole test, each of which was done three times). Ballottement and simultaneous auscultation were positive on the left side. Intestinal motility was poor and there was abdominal guarding. Transrectal examination revealed a loss of negative pressure in the abdominal cavity but the faeces were normal. The California mastitis test was positive in all quarters. The cow had peritarsitis in the left hind leg but was not lame. Urinalysis using a test strip (Combur9^®^, Roche, Basel) produced no abnormal findings (pH = 9; leukocytes, nitrite, urobilinogen, bilirubin, ketone bodies, protein, haemoglobin/erythrocytes negative, mild glucosuria presumably of prior intravenous infusion of a solution containing glucose), and the rumen chloride concentration was 25 mmol/L (normal 15–30 mmol/L, [16]). The main abnormal laboratory findings were hyperproteinaemia (82 g/L, normal 60–80 g/L, [15], leukopenia (4600 cells/µL, normal 5000–10,000 cells/µL), hypokalaemia (2.9 mmol/L, normal 4–5 mmol/L) and hypophosphataemia (0.75 mmol/L, normal 1.3–2.4 mmol/L). The glutaraldehyde test clotting time was 4 min (normal 10–15 min).

**Fig. 1:**
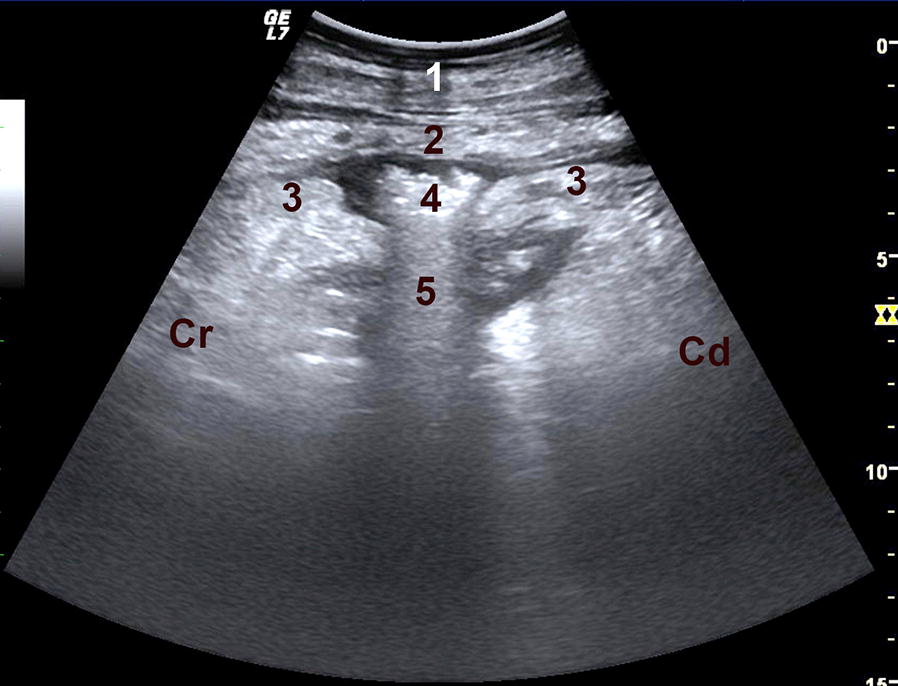
Ultrasonogram of a perforated abomasal ulcer. Ultrasonogram of a perforated (type-3) abomasal ulcer in a Jersey cow. The abomasum is characterised by an irregularity in its contour measuring several centimetres and containing a 2.5 cm (width) × 1.5 cm (depth) hyperechogenic plug-like structure (fibrin clot). A comet-tail artefact is visible medial to the hyperechogenic mass. Echogenic (fibrinous) changes are seen between the abdominal wall and the abomasum. 1 Abdominal wall, 2 echogenic fibrin layer, 3 abomasal wall, 4 hyperechogenic fibrin clot, 5 comet-tail artefact, *Cr* cranial, *Cd* caudal

Based on the positive foreign body tests, abdominal guarding, rumen atony and tympany and reduction in negative abdominal pressure, a tentative diagnosis of peritonitis was made, and the auscultatory findings on the left were suggestive of left displacement of the abomasum (LDA). Ultrasonography of the reticulum and the abdominal cavity [17] and radiography of the reticulum [18] were carried out to confirm the diagnosis and to rule out traumatic reticuloperitonitis. Ultrasonographic findings typical of LDA, which is the interposition of the abomasum between the rumen and the left abdominal wall, were seen on the left side [8, 10]. Furthermore, echogenic changes, which were interpreted as fibrin [18], were seen at one location at the interface between the abdominal wall and the abomasum (Fig. 1). Associated with that lesion was a hyperechogenic plug-like mass, 2.5 cm wide and 1.5 cm deep, which was accompanied medially by an approximately 1.5 to 2.0 cm × 7.0 cm comet tail artefact. Moreover, the abomasal contour was interrupted at this location. Radiographic findings of the reticulum were normal.

**Fig. 2:**
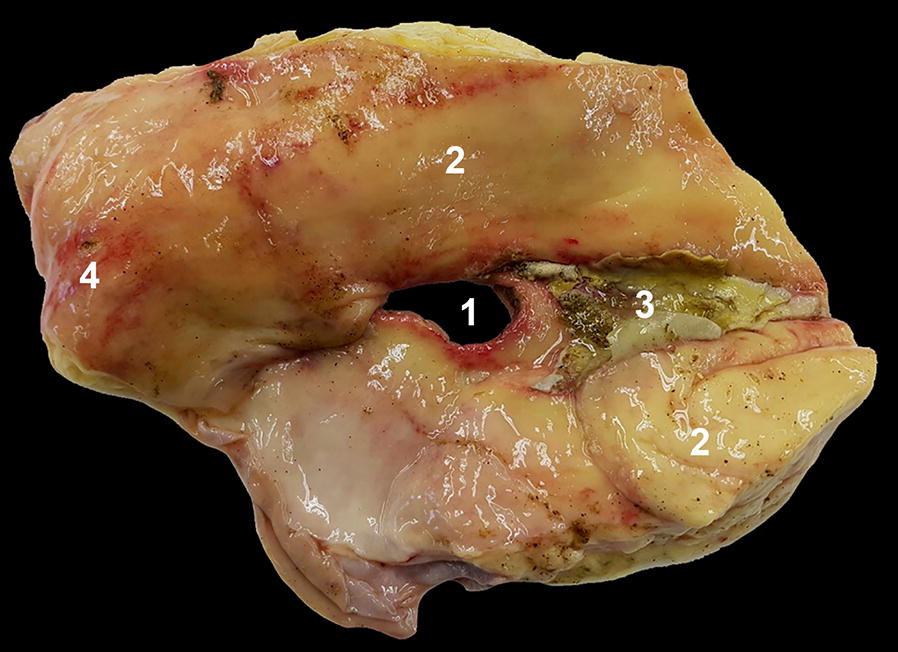
Image of the abomasal ulcer. Image of the mucosal surface of a part of the abomasum from a cow with type-3 abomasal ulcer. A perforated ulcer is visible at the centre and fibrin deposits containing pieces of green ingesta are seen to the right of the perforation. 1 Abomasal perforation, 2 abomasal mucosa with multifocal areas of hyperaemia, 3 fibrin with ingesta, 4 type-1 ulcer

Based on the ultrasonographic findings, a diagnosis of localised fibrinous peritonitis attributable to a perforated (type-3) abomasal ulcer was made, in addition to LDA. Based on a grave prognosis, the cow was euthanased and underwent postmortem examination. Incision of the abdominal cavity resulted in release of malodorous gas, and small amounts of free greenish plant material were seen within the abdominal cavity. The abomasum was in an anatomically normal position at the time of examination and had two perforated ulcers measuring 4.0 and 1.5 cm in diameter, respectively, and multiple small type-1 ulcers (Fig. 2). There were thick fibrinous adhesions on the serosa surrounding the largest ulcer.

## Discussion

The different types of abomasal ulcers have been described in many studies [1–5, 19–24]. Type-3 abomasal ulcers are typically located directly adjacent to other visceral organs and the omentum or parietal peritoneum. This results in localised inflammation and adhesions, which limit the leakage of abomasal contents into the peritoneal cavity [3]. Type-3 ulcers are associated with localised peritonitis and occasionally abscesses, and the clinical signs are similar to those seen in cows with traumatic reticuloperitonitis [19, 21]. The most common clinical signs in 60 cows with type-3 ulcer were, in decreasing order of frequency, complete or almost complete anorexia, abnormal demeanour, reduced skin surface temperature, congested scleral vessels, abdominal guarding, tachypnoea, fever and tachycardia [3], all of which were also seen in our case. Rumen tympany was also present, which was in agreement with the results of another study [25]. Only 10% of 60 cows with type-3 ulcer have been shown to have melena [3], which is consistent with the findings in our case. Hyperproteinaemia, reduced clotting time in the glutaraldehyde test and hypokalaemia were observed in the present case and were seen in 32, 46 and 75% of 60 cows with type-3 ulcer, respectively. Hyperproteinaemia and reduced clotting time indicate inflammation and are typically seen in cows with peritonitis, whereas hypokalaemia is attributable to reduced forage intake [26].

A gap partially occupied by a plug-like clot of fibrin in what otherwise should have been a smooth abomasal contour was clearly imaged by ultrasonography. It was likely that bacterial gas production led to a marked increase in the echogenicity of the fibrin clot and the comet artefact medially. This notion was supported by malodorous gas that escaped after the abdominal cavity was opened. The fibrinous adhesions between the abdominal wall and the abomasum were the result of massive inflammation caused by leakage of abomasal contents. Subjective signs of abomasal ulcers such as lesions associated with peritonitis have been seen via ultrasonography in cattle with type-3 [3], type-4 [4] and type-5 ulcer [5]. For instance, ultrasonographic examination of 46 cows with type-3 ulcer showed fibrin deposits on the abomasal serosa in five cows and free abdominal fluid in the abomasal region in six cows [3]. In the present case, imaging of the ulcer was facilitated by abomasal displacement, which resulted in direct juxtaposition of the ulcer and the abdominal wall and close proximity of the lesion to the ultrasound probe. However, 16 of 60 cows with type-3 abomasal ulcer had LDA, but ultrasonographic visualisation of the ulcer was not possible in any of them; nevertheless, the detection of signs of peritonitis in the cranial abdomen were strongly suggestive of type-3 ulcer particularly in the absence of radiographic evidence of a foreign body [3]. This appears to be the first time that ultrasonography could be used for the antemortem diagnosis of type-3 abomasal ulcer rather than relying on exploratory surgery [25] and/or a postmortem examination [3].

We were surprised to find the previously displaced abomasum in its correct position during postmortem examination considering the degree of peritonitis and the amount of fibrin between the left abdominal wall and the perforated ulcer. We suspected that the adhesions were at an early stage of development and that tearing occurred during movement of the dead cow from the barn to the postmortem room.

## Conclusion

This case report should encourage clinicians to continue to place emphasis on ultrasonographic examination of the abdomen when perforated abomasal ulcers are suspected, particularly in cows with LDA. Advances in ultrasound technology and the availability of high-resolution transducers have increased the probability of detecting a variety of disorders including ulcers.
